# Retraction: Prenatal Cocaine Exposure Uncouples mGluR1 from Homer1 and Gq Proteins

**DOI:** 10.1371/journal.pone.0266628

**Published:** 2022-03-30

**Authors:** The PLOS ONE Editors

Following the publication of this article [1], concerns were raised regarding results presented in Figs 2 and 5. Specifically,

- In the Fig 2A Frontal cortex Homer1 panel, there appears to be a horizontal discontinuity in the background above the bands in lanes 1 and 2, as well as horizontal and vertical discontinuities around the top band in lane 4.
- The band in the fourth lane of the Fig 2A Frontal cortex mGluR1 panel appears similar to the band in the first lane of the Fig 5A Frontal cortex mGluR1 panel.
- The bands in lanes 2 and 3 of the Fig 2A Hippocampus mGluR1 panel appear similar to the bands in the Fig 5A Hippocampus mGluR1 panel.

The corresponding author disagrees with the concerns raised with Figs 2 and 5. Regarding the irregularities in the Fig 2A Homer 1 panel, the corresponding author suggests that the observed irregularities are likely the result of image artifacts or experimental artifacts such as reagent remnants or patches intrinsic to the membrane. Furthermore, the corresponding author stated that the mGluR1 panels presented in Figs 2A and 5A were obtained from separate blots.

The corresponding author provided image data to support their published result in this [1] and other PLOS ONE articles [2–5]. Per PLOS’ assessment of the data files, the pixel patterns in background areas of blot images provided for multiple panels in [1–5] appear more similar than would be expected for data obtained in independent experiments. The corresponding author stated that the repetitive features in the background noise of the underlying data are likely the result of scanner artifacts.

The data and comments provided to PLOS did not resolve the concerns about the integrity and reliability of the reported data. In light of these issues, the *PLOS ONE* Editors retract this article.

HYW did not agree with the retraction and stands by the article’s findings. KB, RP, SKG, MW, and EF either did not respond directly or could not be reached.
